# Current Situation of Mycotoxin Contamination and Co-occurrence in Animal Feed—Focus on Europe

**DOI:** 10.3390/toxins4100788

**Published:** 2012-10-01

**Authors:** Elisabeth Streit, Gerd Schatzmayr, Panagiotis Tassis, Eleni Tzika, Daniela Marin, Ionelia Taranu, Cristina Tabuc, Anca Nicolau, Iuliana Aprodu, Olivier Puel, Isabelle P. Oswald

**Affiliations:** 1 BIOMIN Research Center, Technopark 1, 3430 Tulln, Austria; Email: elisabeth.streit@biomin.net; 2 Faculty of Veterinary Medicine, Aristotle University of Thessaloniki, St Voutyra 11, 54627 Thessaloniki, Greece; Email: tassisp@gmail.com (P.T.); eltzika@vet.auth.gr (E.T.); 3 National Institute for Research and Development in Animal Biology and Nutrition (IBNA), Calea Bucuresti, 077015 Balotesti, Romania; Email: daniela.marin@ibna.ro (D.M.); ionelia.taranu@ibna.ro (I.T.); cristina.tabuc@ibna.ro (C.T.); 4 “Dunarea de Jos” University of Galati, Domneasca 47, 800008 Galati, Romania; Email: anca.nicolau@ugal.ro (A.N.); iuliana.aprodu@ugal.ro (I.A.); 5 Institut National de la Recherche Agronomique (INRA), UMR1331, Toxalim, Research Centre in Food Toxicology, 180 chemin de tournefeuille, F- 31027 Toulouse cedex 3, France; Email: olivier.puel@toulouse.inra.fr (O.P.); ioswald@toulouse.inra.fr (I.P.O.); 6 Université de Toulouse, INP, UMR1331, Toxalim, F- 31000 Toulouse, France

**Keywords:** mycotoxins, feed, co-occurrence, Europe, aflatoxin, deoxynivalenol, zearalenone, fumonisins, ochratoxin A, T-2

## Abstract

Mycotoxins are secondary metabolites produced by fungi especially those belonging to the genus *Aspergillus*, *Penicillum* and *Fusarium*. Mycotoxin contamination can occur in all agricultural commodities in the field and/or during storage, if conditions are favourable to fungal growth. Regarding animal feed, five mycotoxins (aflatoxins, deoxynivalenol, zearalenone, fumonisins and ochratoxin A) are covered by EU legislation (regulation or recommendation). Transgressions of these limits are rarely observed in official monitoring programs. However, low level contamination by *Fusarium* toxins is very common (e.g., deoxynivalenol (DON) is typically found in more than 50% of the samples) and co-contamination is frequently observed. Multi-mycotoxin studies reported 75%–100% of the samples to contain more than one mycotoxin which could impact animal health at already low doses. Co-occurrence of mycotoxins is likely to arise for at least three different reasons (i) most fungi are able to simultaneously produce a number of mycotoxins, (ii) commodities can be contaminated by several fungi, and (iii) completed feed is made from various commodities. In the present paper, we reviewed the data published since 2004 concerning the contamination of animal feed with single or combinations of mycotoxins and highlighted the occurrence of these co-contaminations.

## 1. Introduction

The term mycotoxin designates a chemically diverse group of secondary fungal metabolites [1,2,3], mainly produced by species of the *Aspergillus*, *Penicillium* or *Fusarium* genus [4,5]. Depending on classification, 300–400 mycotoxins are known today [6]. Contamination may occur in the field as well as during (improper) storage and is largely dependent on environmental factors [7,8]. When ingested by humans or animals above a certain concentration, mycotoxins will cause a toxic response referred to as mycotoxicosis [1,4,5]. The symptoms elicited by mycotoxin consumption range from reduced animal productivity (reduced body weight gain, reduced fertility) and immune suppression [9], resulting in increased susceptibility to diseases and parasites to overt disease and death. Clinical symptoms of mycotoxin intoxication include diarrhoea, liver and kidney damage, pulmonary oedema, vomiting, haemorrhaging and tumours [1,7]. Under field conditions, mycotoxins usually occur in concentrations leading to reduced animal performance and/or immune suppression without causing any obvious clinical symptoms [5,8]. It is important to emphasise that it is very common for an array of mycotoxins to occur together at low concentrations. This is on the one hand due to the ability of various fungi to simultaneously produce a variety of mycotoxins [10,11] and on the other hand due to the fact that any given commodity is likely to be infected with different types of fungi. Moreover, compound feed is made up of a number of different commodities contributing to the final mycotoxin profile. 

In the feed manufacturing process, only a limited number of mycotoxins are subject to regular testing and legal regulations/guidance [12,13], namely aflatoxins (AF), deoxynivalenol (DON), zearalenone (ZEN), fumonisins (FB) and ochratoxin A (OTA). Consequently, this review will focus on these contaminants. In addition, the case of T-2/HT-2 toxin will be addressed. First, a brief introduction about each of the aforementioned toxins including associated adverse effects will be provided. Subsequently, an overview of their occurrence and co-occurrence, with special focus on the EU, will be given.

## 2. Major Mycotoxins

### 2.1. Aflatoxins

Aflatoxins (B_1_, B_2_, G_1_ and G_2_) are considered to be the group of mycotoxins of greatest concern from a global perspective [4]. They are primarily produced by *Aspergillus flavus*, *A. parasiticus *and in rare cases, by *A. nomius* [14]. AFB_1_, the most abundant and most toxic aflatoxin [15], is often referred to as the most potent naturally occurring carcinogen [4]. It is classified as a Group 1 human carcinogen by the International Agency for Research on Cancer (IARC) [16]. Lactating animals fed AFB_1_ contaminated diets will produce milk contaminated with its monohydroxylated derivative AFM_1_ [15], classified as 2B, possibly carcinogenic to humans [16].

Aflatoxin production occurs primarily in regions with tropical or subtropical climates. Hence, from a European perspective, imported feed such as peanut cake, palm kernel, copra and corn gluten meal (depending of origin) is considered to be the most common source of exposure [15]. However, the EFSA report also cites findings from Italy reporting the detection of AFB_1_ in maize originating from the Po valley after a growth period characterized by high temperatures, drought and strong insect damage. As a result, regional milk samples collected after this particular harvest were found to be contaminated with AFM_1_ concentration exceeding EU limitations. The incident occurred in 2003 and is discussed in more detail in the section on mycotoxin occurrence in Europe.

The main target organ of aflatoxin toxicity is the liver [15]. Long term exposure of animals to subacutely toxic levels of AFs is associated with liver lesions and/or tumours [17], inferior egg shell and carcass quality, increased disease susceptibility [5], reduced feed efficiency [18], and teratogenicity [1].

### 2.2. Deoxynivalenol

Deoxynivalenol (DON) belongs to the trichothecene group and, albeit being one of its least acutely toxic members, is of particular interest owing to its high prevalence [19]. More precisely DON is classified as type-B trichothecene [20]. It is produced by *Fusarium culmorum* and *F. graminearum* [10,19]. DON contamination is observed worldwide, with cereal crops such as wheat, maize or barley being most frequently affected [20]. Furthermore, silage contamination is regularly observed [21]. Cold and wet weather conditions favour DON production [19] and it was found that the timing of the rainfall is more influential than the amount of precipitation [20,22].

In animal husbandry, DON, also known as vomitoxin, is primarily known for causing feed refusal and emesis in pigs [7]. This mycotoxin also alters the immune response [9,23] and the intestinal functions [24]. DON may be produced together with two acetylated derivatives, 3-AcDON and 15-AcDON, that have differential toxicity on pig intestine [25]. Poultry are not as sensitive to DON [26] and feed refusal is only observed at very high concentrations (16–20 mg/kg feed) [20]. Ruminants are the least sensitive animal species to DON, a fact that is attributed to the capacity of rumen microflora to detoxify this mycotoxin [19].

### 2.3. T-2 Toxin and HT-2 Toxin

T-2 and HT-2 toxin are two of the most toxic members of the trichothecene group. They belong to the type A-trichothecenes and are produced by *F. sporotrichioides*, *F. poae* and other *Fusarium* species [27,28]. Oats and oat products were found to be particularly prone to contamination with high levels of T-2 and HT-2 [29] followed by barley [27]. 

Toxicity data on T-2 and HT-2 are notoriously scarce compared to the other mycotoxins addressed in this review. They were found to impair protein synthesis and exert immunotoxic, haematotoxic and myelotoxic effects [29,30]. Dietary exposure to T-2 and HT-2 was reported to weaken acquired immune response in pigs [31] and cause oral lesions in poultry [32,33]. Feed intake depression and reduced weight gain have been observed in both species [32,33,34]. Higher T-2/HT-2 concentrations were furthermore found to negatively influence egg production and egg shell thickness in laying hens [35,36]. Pigs are most sensitive to T-2/HT-2, followed by poultry. Ruminants are again protected by their microflora and found to be least sensitive to these toxins [29]. 

A comprehensive review on the occurrence of T-2/HT-2 in Europe was provided by van der Fels-Klerx [27]. As a matter of fact, reports on T-2 and HT-2 occurrence are largely restricted to Europe, most often coming from the northern parts of the continent like Scandinavia or the UK [27,28]. When guidance values were set for the other major mycotoxins in feed in 2006 by the European Commission data on T-2/HT-2 were deemed too scarce for setting scientifically sound guidance values and further monitoring was recommended [13]. In a recent opinion piece, the EFSA panel on contaminants in the food chain (CONTAM) concluded that based on available data the animal health risk from dietary exposure to T-2/HT-2 was low [29]. 

### 2.4. Zearalenone

Like DON, zearalenone (ZEN) is produced by fungi of the *Fusarium* genus. *F. culmorum*, *F. graminearum* and *F. heterosporum* are among the species found to produce ZEN [10]. Again the risk of contamination is highest in cereal crops [37] but silages, forage, and straw are also likely to contain ZEN [7,21].

Acute toxicity of ZEN is low and adverse effects observed are caused by its ability to interact with the oestrogen receptor [37]. Consequently ZEN is mainly associated with fertility problems and hyper oestrogenic symptoms such as swelling of the vulva and uterus enlargement [1,5,38]. Female swine are most sensitive to ZEN exposure [37] whereas poultry are found to be very tolerant [38]. It is very unlikely that naturally contaminated feedstuff contains ZEN at concentrations sufficiently high to cause adverse effects in poultry. Data on dairy cows is limited but also suggests low responsiveness to ZEN [37].

### 2.5. Fumonisins

Fumonisins are also counted among the *Fusarium* mycotoxins. In feed crops they are most commonly produced by *F. proliferatum* and *F. verticillioides*. Of the numerous fumonisin analogues known, the B series (FB_1_, FB_2_, and FB_3_) is most important regarding occurrence and toxicity. FB_1_ is of greatest concern, as it is the most prevalent and the most toxic of the fumonisins [39]. It has been classified as 2B, possibly carcinogenic to humans [40]. Fumonisin contamination is commonly associated with maize and maize products [39,41]. In a wider context the classification of fumonisins as *Fusarium* mycotoxins is no longer valid as recently black Aspergilli, most notably *A.* *niger*, were also found capable of producing fumonisins [42]. They were reported to cause severe contamination of dried grapes with FB_1–4_ and other fumonisin isomers [43].

Fumonisin contaminated feed will cause severe diseases like pulmonary oedema in swine and leukoencephalomalacia in horses [44]. Furthermore, fumonisins were found to be immunosuppressive [5,45,46], hepatotoxic, and nephrotoxic [1]. 

Fumonisins, DON and ZEN are considered to be the most important exponents of the *Fusarium* mycotoxins with regard to animal health implications [41,47] and associated economic loss [48]. Although fumonisin contamination is not very common in crops other than maize, *Fusarium* mycotoxins in general are often found to occur together in contaminated cereals [20,41,47]. In most cases, the resulting toxic effects will be additive combinations of the mycotoxins’ individual toxicities but synergistic interactions have been observed [47,49].

### 2.6. Ochratoxin A

Ochratoxin A contamination is predominantly associated with insufficient drying or improper storage. It is found all over the world. In temperate regions, OTA contamination is mostly due to *Penicillium verrucosum* infection while *Aspergillus* species such as *A. carbonarius* account for OTA production in warmer regions [50,51]. As for feed ingredients, OTA is most frequently found in cereals but is also known to contaminate soy beans and peanuts. Since fungal growth often occurs at localised hot spots (*i.e.*, an area of elevated water activity) in the stored grain, OTA distribution in contaminated feed lots tends to be very heterogeneous. This fact poses a particular challenge when testing for OTA contamination [50]. Ochratoxin A has been classified as possibly carcinogenic to humans (Group 2B) [52].

The primary target organ for OTA toxicity is the kidney [51]. OTA contamination has been linked to outbreaks of nephropathy in pigs and poultry. It is furthermore associated with immunosuppression, reduced growth rate and increased mortality [50,51]. Owing to their rumen microflora’s ability to degrade OTA to the less toxic ochratoxin α, ruminants are less sensitive to OTA. However, negative effects on milk production have been described [51]. With regards to OTA residues in animal derived food, specialities made from porcine blood are of most concern. Accumulation in kidney and liver was also observed although at a lesser extent. However, it is estimated that animal derived foodstuff only accounts for 3%–10% (depending on eating habits) of human dietary exposure to OTA in Europe [50].

## 3. EU Regulations and Guidance Values

In the European Union, maximum levels for aflatoxin B_1_ in feedstuff have been established by the European Parliament [53] and guidance values for DON, ZEN, FB and OTA were issued by the Commission [13]. Selected examples are given in Table 1. Legally binding regulations (as opposed to mere guidance values) have been issued for AFB_1_ as this mycotoxin is particularly prone to carry-over in milk (AFM_1_). The maximum level is designed to prevent the occurrence of AFM_1_ at levels considered harmful to human health.

## 4. Occurrence of Mycotoxins in European Feed and Feed Raw Materials

A number of studies on the occurrence of mycotoxins in European feedstuffs have been published. Examples are given in Table 2. As this review aims at providing an update on mycotoxin contamination in feed, only publications dating from 2004 or later were included in this table. The data clearly show that mycotoxins are ubiquitously present in feed material throughout Europe and that maximum contamination levels exceeding the EU maximum levels or guidance values are likely to occur.

**Table 1:** Selected examples of maximum levels for aflatoxin (AFB_1_) (European Parliament 2002) and guidance values for deoxynivalenol (DON), zearalenone (ZEN), fumonisins (FB) and ochratoxin A (OTA) (European Commission 2006) in feed.

Mycotoxin	Feedstuff	Maximum Level or Guidance Value ^a^ [µg/kg]
Aflatoxin B_1_	compound feed for dairy animals and young animals feed materials	5
		20
Deoxynivalenol	complementary and complete feedingstuffs for pigs	900
	cereals and cereal products	8,000
	maize by-products	12,000
Zearalenone	complementary and complete feedingstuffs for piglets and gilts	100
	complementary and complete feedingstuffs for calves, dairy cattle, sheep and goats	500
	maize by products	3,000
Fumonisins B_1_ + B_2_	complementary and complete feedingstuffs for pigs, horses, rabbits and pet animals	5,000
	maize and maize products	60,000
Ochratoxin A	complementary and complete feedingstuffs for pigscereals and cereal products	50250

^a ^moisture content: 12%.

Miraglia *et al*. [54] emphasised that occurrence patterns of mycotoxins in Europe are expected to change as a consequence of rising average temperatures. Southern Europe is used as an example to support this statement. The authors report that while the importance of DON is about to decrease, *A.* *flavus* infection and aflatoxin contamination, previously uncommon in Europe, will become increasingly important. In fact, in 2003 a hot and dry growing season led to severe *A.* *flavus* infection of maize in northern Italy [55]. A survey on 110 samples showed an AFB_1_ incidence of 75% with a mean contamination of 4.4 µg/kg. Using this maize as feedstuff for dairy cattle led to a widespread AFM_1_ contamination in milk and several thousand tons of milk exceeding the EU legal limit of 0.05 µg/kg AFM_1_ [12] had to be discarded. The study by Decastelli *et al.* [56] presents the results of a surveillance plan spanning the two years following this incident (Table 2). Indicators of this impending change were also found by Goertz *et al.* [57], who reported *F.* *verticillioides*, commonly associated with warmer and drier regions such as Italy or Spain, to be the predominant *Fusarium* species isolated from maize grown in Germany in 2006. As a result, FB contamination was detected in 34% of the samples (Table 2). According to the weather data provided in the report, July and September 2006 had been particularly warm and dry all over Germany.

**Table 2:** Occurrence of Mycotoxins in European feed and feed raw materials—surveys published since 2004. Mycotoxins other than those discussed in the introduction are listed for the sake of completeness, maximum contamination levels are listed in the “range”-column if no information on the contamination range was provided in the respective publication, exponents next to the mycotoxins’ names indicate the analysis method.

Country	Commodity	Mycotoxins	*n*	% positives	Range (µg/kg)	Reference
EU(CZ, DK, ES, PT, HU)	sows feed, wheat, maize	23 mycotoxins	82	82		Monbaliu *et al.* [58]
		e.g., DON ^z^	82	63	74–9,528	
		FB_1_	82	44	36–5,114	
		ZEN	82	15	58–387	
		HT-2	82	9	22–116	
		OTA	82	2	22–33	
Europe	feed materials	AF ^x,y^	169 ^a^	15	max: 103	Rodrigues and Naehrer [21]
		ZEN	1517	20	1,045	
		DON	2036	58	49,000	
		FB	114	48	11,050	
		OTA	192	35	331	
Southern Europe	feed materials	AF ^x,y^	127 ^a^	25	0.5–66	Griessler* et al.* [59]
		ZEN	303	28	10–2,939	
		DON	348	66	52–4,827	
		T-2/HT-2	65	8	35–137	
		OTA	46	22	1–54	
		FB	89	66	25–36,390	
Portugal	dairy cow’s feed	AF ^y^	1001	37	1–74	Martins *et al.* [60]
Portugal	feed and raw materials	AF ^y^	1936	26	1–80	Martins *et al.* [61]
		DON ^w^	515	6	100–1,649	
		FB ^y^	545	4	10–40	
		ZEN ^w^	30	13	104–356	
Portugal	feed fattening pigs	OTA ^y^	277	8	2–6.8	Almeida *et al.* [62]
		ZEN	277	25	5–73	
		DON	277	17	100–864	
	sows feed	ZEN	127	30	5–58	
		FB	127	9	50–391	
Spain	feed and raw materials	OTA ^y^	91	30	0.1–12	Jaimez *et al.* [63]
		ZEN	91	8	0.5–2	
Spain	Barley	AF ^y^	123	100	max: 0.8	Ibáñez-Vea *et al.* [64]
		OTA	123	58	4	
		ZEN	123	39	19	
		DON ^v^	123	95	1,111	Ibáñez-Vea *et al.* [65]
		NIV	123	20	1,435	
		FUS-X	123	2	17	
		15-AcDON	123	57	65	
		3-AcDON	123	28	20	
		DAS	123	25	2	
		T-2/HT-2	123	24	533	
Italy	feed for dairy cows	AFB_1_^x,y^	616	8 (in 2004)	not specified	Decastelli *et al.* [56]
				0 (in 2005)		
Croatia	maize^b^	DON ^w,x,y^	40	85	15–17,920	Pleadin *et al.* [66]
		ZEN	40	88	2–5,110	
Croatia	maize	FB_1_^y^	49	100	142–1,378	Domijan *et al.* [67]
		FB_2_	49	6	68–3,084	
		ZEN	49	84	0.4–39	
		OTA	49	39	0.9–3	
Greece	feed and raw materials	AFB_1_^w^	119	0		Vlachou *et al.* [68]
			183	4	10–90	
Czech Republic	grass(field trial)	ZEN ^x^			max: 173	Skládanka *et al.* [69]
		DON			71	
		FB			Nd	
		AF			nd	
Slovakia	poultry feed	FB ^y^	50	98	36–1,160	Labuda *et al.* [70]
		moniliformin	50	52	42–1,214	
		ZEN ^u, y, z^	50	88	3–86	Labuda *et al.* [71]
		DON	50	56	64–1,230	
		T-2	50	90	1–130	
		HT-2	50	76	2–173	
		3‑AcDON,				
		15‑AcDON,				
		NIV and DAS also analysed				
Bulgaria	barley, wheat, maize	ZEN ^y^	91	8	max: 148	Manova and Mladenova [ 72]
		FB	19^c^	95	342–4,050	
Romania	Maize, wheat,	AFB1x	86	38	max: 52	Tabuc et al. [ 73]
	barley, oat,	OTA	86	14	81	
	soya,	DON	86	70	2,248	
	sunflower,	ZEN	86	32	496	
	colza, rice,	FB	86	41	1,008	
	triticale, rye		86			
			86			
Romania	wheat	DON	40	43	19 –96	Banu *et al.* [74]
		ZEN	40	10	3–6	
Poland	cereals, corn, mixed feed, silages	AF ^y, z^	1255	3–10	0.2–0.9	Grajewski *et al.* [75]
		OTA	1255	36–87	28–760	
		FB	1255	60–91	435–9,409	
		ZEN	1255	60–87	422–1,150	
		DON	1255	83–95	3,090–14,470	
		NIV, T-2 and HT-2 also analysed		range of % positives/year	range of yearly maxima	
Germany	Maize	32 ^y^			max:	Goertz *et al.* [57]
	(2006 and 2007 harvest)	e.g. DON (2006)	44	75	19,570	
		ZEN (2006)	44	27	860	
		FB_1_ (2006)	44	34	20,690	
		DON (2007)	40	90	16,250	
		ZEN (2007)	40	93	14,580	
		FB_1_ (2007)	40	0	50 (LOQ/2)	
Switzerland	maize(field trial)	DON ^x, z^	19^d^	100	210–8,580	Dorn *et al.* [76]
		ZEN	19	79	16–1,260	
		FB	19	10	1,180–2,110	
		NIV	19	36	80–1,300	
	growers fields	DON	12	92	160–8,570	
		ZEN	12	92	60–2,240	
		FB	12	17	300–265,000	
		NIV	12	25	440–1,530	

^a^ number of samples analysed for each mycotoxin, total number of samples 416; ^b ^sampling after exceptionally cold and wet 2010 growing season; ^c^ FBs were determined in maize only; ^d^ 19 samples in 2006, 2007 samples not shown; ^u ^GC;^ v ^GC-MS;^ w^ TLC;^ x^ ELISA; ^y^ HPLC; ^z^ HPLC-MS/MS; CZ: Czech Republic; DK: Denmark; ES: Spain; PT: Portugal; HU: Hungary; NIV: Nivalenol; FUS-X: Fusarenon-X; DAS: Diacetoxyscirpenol; nd: not detected.

It is however difficult to infer trends or recent developments regarding mycotoxin contamination in European feed from the data presented in Table 2. There are a number of reasons for this, not the least of which is the strong influence of the respective cropping season’s climate on the contamination level, causing a high year to year variation of the results, which underlines the importance of implementing regular surveillance programs. Also, applied analysis methods were diverse, including ELISA, TLC, GC and HPLC. Moreover sampling methods are often not described in detail, yet, sampling is considered the largest source of error in mycotoxin analysis [77]. 

## 5. EU and National Monitoring

The vast majority (>98%) of mycotoxin related entries concerning feed in the RASFF (Rapid Alert System for Food and Feed) database [78] report the detection of aflatoxins. This is not surprising, as aflatoxin B_1_ is to date the only mycotoxin for which legal maximum levels in feedstuff have been established in the European Union. In general, mycotoxins only account for a rather small fraction of the feed related entries in the RASFF database [79], probably for the same reason.

Austrian feed and feed raw materials generally show good compliance with EU regulations and guidance levels. DON is detected most often (around 60% positives in cereal samples other than maize and 95% positives in maize samples). ZEN is the second most common mycotoxin, found in about 70% of unprocessed maize kernel samples. It is rarely detected in other cereal samples. Other mycotoxins such as FB and OTA are detected much less frequently, FB for example is only quantifiable in 10%–30% of each year’s maize samples [80,81,82]. One Austrian maize sample in 2009 and one in 2010 were found to exceed the guidance level for DON [80,81].

The vast majority of the feed samples analysed for mycotoxin contamination in 2010 within the scope of German official controls proved to be compliant with legal requirements. Seven oilseed samples were found to contain AFB_1_ above the maximum limit. As 1810 samples were tested for AFB_1_, this makes for only 0.4% of objectionable samples [83]. Two samples exceeded the guidance value for DON and three samples contained OTA levels above the respective guidance value [84].

Feed official control system in Greece reported a high compliance to EU regulation 1881/2006 in the majority of samples tested in 2010. For the detection of aflatoxin B_1_, 71 feed and grain samples (wheat, maize, barley *etc.*) were tested and 5 were non-compliant to EU regulation 32/2002. All samples analysed for OTA, ZEN, and DON were compliant to the respective legislation and no excess levels of T-2 toxin were found either [85].

RIKILT Institute for Food Safety published a report analysing the results from Dutch official controls of feed from 2001–2009 [86]. Mycotoxin concentrations are generally well below the respective maximum level or guidance values although some individual samples exceeded admissible limits. Contamination levels in cereals were generally stable. High DON concentrations were found in liquid pig feed samples, 10% of which exceeded the guidance value. Oil seeds (sunflower, groundnut) and rice were reported to occasionally contain AFB_1_ concentrations above the regulatory limit. It was also noted, that soy bean samples with high ZEN content often originated from Argentina.

In Sweden, all feed samples analysed under the national monitoring scheme in 2010 were compliant with EU regulations [87]. In fact, the Swedish board of agriculture did not detect any transgressions of mycotoxin maximum levels or guidance values since 2006 [88,89,90]. In early 2006, aflatoxin contaminated rice meal used in dairy cattle feed production had been identified as the cause of elevated AFM_1_ levels in Swedish milk [91].

## 6. Mycotoxin Co-occurrence

Considering the fact that mycotoxigenic fungi are usually capable of producing more than one mycotoxin [4,10,11] and that feed raw materials are commonly infected with various fungal species at a time [10], studying the occurrence of any given mycotoxin alone provides incomplete information about the risk associated with the respective feedstuff. Compound feed is particularly vulnerable to multiple contaminations as it typically contains a mixture of several raw materials [92]. In their meta-analysis, Grenier and Oswald [49] reviewed 112 publications on toxicological interactions of mycotoxins. They found that most of the studies reported synergistic or additive interactions regarding adverse effects on animal performance. When it comes to other parameters, especially biochemical ones, results were more variable, ranging from synergistic to antagonistic for the same toxin combination and target variable. Nevertheless the summarised findings underline the importance of studying mycotoxin co-occurrence. 

Stoev *et al.* [93] arrived at a similar conclusion. They analysed a total of 50 feed samples from Bulgarian pig and poultry farms experiencing nephropathy problems. They found that, although all samples were contaminated with OTA at a mean concentration exceeding the EC guidance levels for pig and poultry feed (50 µg/kg and 100 µg/kg respectively) [13], OTA concentration alone was not high enough to explain the observed symptoms. It was inferred that the nephropathy problems were caused by the combined effects of OTA, FB_1_ and penicillic acid. Furthermore, a hitherto unknown metabolite found in 92% of the samples, is suspected as an additional causative agent. 

Both publications highlight the necessity of testing for an array of mycotoxins in order to accurately determine feed quality and potential risks. This section is aimed at providing an overview of mycotoxin co-occurrence found in feed and feed raw materials with special focus on European data. The findings mentioned in the following paragraphs are also summarised in Table 3 and Table 4. Table 4 contains data on mycotoxin co-occurrence in Europe while Table 3 provides additional information concerning the co-occurrence of AF and FB in maize from South America. Maize is particularly prone to infection by mycotoxigenic fungi, most notably *Fusarium* sp. [94]. Therefore it is often subject to studies on mycotoxin contamination and co-occurrence.

As for Europe, an UK study screening maize products intended for animal feed for 22 mycotoxins found that all 67 samples were co-contaminated with up to 12 different *Fusarium* mycotoxins [95]. FBs and DON occurred together in 75% of the samples and 15-AcDON, moniliformin, and ZEN were frequent co-contaminants. None of the samples contained detectable amounts of AF. Prior to this study, Scudamore *et al.* [96] conducted a survey on 330 samples of feed ingredients and found that maize was by far the most affected by co-contamination. In total, 60% of the maize samples tested positive for more than one mycotoxin, AF and FB being the most common combination (28%). Another publication reported the occurrence of 14 different *Fusarium* mycotoxins in 84 German maize samples sourced after the 2006 and 2007 harvest [57]. Although no information regarding co-occurrence is provided, the high individual incidences of several mycotoxins imply co-occurrence of DON (and its acetylated forms), ZEN, moniliformin, beauvericin, nivalenol, enniatin B, FBs, and/or HT-2 toxin. Likewise, co-occurrence of trichothecenes with ZEN and OTA in 760 Hungarian maize samples can be deduced from the individual toxin prevalences reported by Rafai *et al.* [97]. This study also comprised 921 samples of other feed raw materials such as wheat, barley, soybean and so on. Typically, DON was found to be the major contaminant, frequently co-occurring with ZEN. 

Several groups investigated the occurrence of aflatoxins and fumonisins in Brazilian maize. The results are summarised in Table 3. Joint contamination with these toxins is particularly concerning as there is evidence that FB_1_ synergistically promotes liver tumours initiated by AFB_1_ [49]. Brazil is the third most important producer of maize in the world [98]. Hence, contamination levels of Brazilian maize are of international significance. Table 3 also lists a long-term study from Argentina, fourth most important producer of maize worldwide [98].

**Table 3:** Summary of the studies reporting mycotoxin co-occurrence in South American maize.

Country	Mycotoxins	*n*	Co-Occurrence	Reference
Brazil	AFB_1_, FB_1_, ZEN	214	AFB_1_, FB_1_: 38%	Vargas *et al.* [99]
			FB_1_, ZEN: 30%	
			AFB_1_, FB_1_, ZEN: 8%	
Brazil	AFB_1_, FB_1_	110	AFB_1_, FB_1_: 54.5%	Carmagos *et al.* [100]
Brazil	AFs, FBs	300	AFs, FBs: 8%	Moreno *et al.* [101]
Brazil	AFs, FBs	200	AFs, FBs: 7%	Rocha *et al.* [102]
Argentina	AF, FB, ZEN, DON	3246	AF, FB: 8.3%	Garrido *et al.* [103]
			FB, ZEN: 2%	

A survey on feed and feed ingredients (*n* = 416) sourced in Southern Europe from January 2005 to August 2009 established that 22% of the compound feed samples contained more than one mycotoxin [59]. It was furthermore reported that 23% of all samples from Spain and 32% of the Italian samples contained at least two mycotoxins. The samples were tested for AFs, ZEN, B-trichothecenes (DON and AcDON), A-trichothecenes (T-2 toxin and HT-2 toxin), FBs, and OTA. It was stated that *Fusarium* mycotoxins, namely type B-trichothecenes, ZEN, and FBs, were major contaminants and that their co-occurrence was frequently observed. Almeida *et al.* [62] analysed 277 samples of feed for fattening pigs marketed in Portugal for OTA, ZEN, and DON. In total, 10% of the samples were found to contain any two-toxin combination, ZEN and DON co-occurring most often. One sample was contaminated by all three toxins. Moreover, concentrations of FBs and ZEN were determined in 127 samples of sows feed, two of which (1.5%) were positive for both analytes. Another study analysing 82 samples of sows feed, wheat and maize sourced in different EU countries in 2008 for the presence of 23 different mycotoxins found that 75% of the samples contained more than one mycotoxin [58]. The majority of these samples contained DON, AcDON, and FBs in combination with other mycotoxins. Of 50 Slovakian poultry feed samples tested for trichothecenes and ZEN, 84% contained more than one mycotoxin. A four toxin combination of DON, ZEN, T-2, and HT-2 was detected most frequently (32%) [71]. A similar study on the occurrence of FB_1_, FB_2,_ and moniliformin in poultry feed from Slovakia found that 25 (50%) of the samples contained all three mycotoxins [70]. The description of the samples in both publications strongly suggests, that the samples analysed in both studies were identical. Merging the results shows that all samples were contaminated with at least three mycotoxins and that 82% contained five or more. The most common combinations being DON, ZEN, T-2, HT-2, FB_1_, and FB_2_ and DON, ZEN, T-2, HT-2, FB_1_, FB_2_, and moniliformin, found in 14% and 12% of the samples respectively. Upon investigating the diets of dairy cattle in the Netherlands, Driehuis *et al.* [104] found that DON and ZEN were co-occurring in 44% of the diets, silage and compound feed being the major sources of exposure. 

Other European studies have focused on a single commodity. For example, Ibáñez-Vea *et al.* [64] tested 123 Spanish barley samples from the 2007 and 2008 harvest for AFs, ZEN, and OTA. Detectable levels of AFB_1_ were reported for all the samples and it was found to co-occur with OTA in 31% of the samples. Co-occurrence of AFB_1_ and ZEN was 12% and AFB_1_, ZEN, and OTA co-contamination was detected in 27% of the samples. The authors published a second study on the occurrence of type A and B trichothecenes in these samples [65]. DON was present in 95% of them and 43% contained three or more trichothecenes. Combining the results of both studies 96% of the samples were found to be contaminated with three or more mycotoxins [105]. The combinations AFB_1_, OTA and DON and AFB_1_, OTA, DON, and ZEN were most frequent, being observed in 29% and 26% of the samples respectively. 

**Table 4:** Overview of studies on mycotoxin co-occurrence in feed and feed ingredients from EU countries.

Country	Commodity	Mycotoxins	*n*	Co-Occurrence	Reference
UK	Maize products	22 different mycotoxins	67	100% contained >1 mycotoxin	Scudamore *et al.* [95]
				up to 12 mycotoxins found to co‑occur	
UK	feed ingredients	AFs, FBs, OTA, ZEN, citrinin, sterigmatocystein		14%, most often in maizeAF, FB in maize most common: 28%	Scudamore *et al.* [96]
Germany	Maize	32 mycotoxins	84	not specified, implied by high individual incidencese.g., DON and ZEN	Goertz *et al.* [57]
Hungary	feed raw materials	8 Fusarium mycotoxins	1681	not specified, implied by high individual incidencese.g., trichothecenes, ZEN and OTA	Rafai *et al.* [97]
Southern Europe	feed and feed ingredients	AFs, ZEN, DON, AcDON, T-2, HT‑2, FBs, OTA	416	22% in compound feed	Griessler *et al.* [59].
				B trichothecenes, ZEN and FB co-occurred frequently	
Portugal	swine feed(fattening)	OTA, ZEN, DON	277	10% contained any two toxins(most often ZEN and DON) OTA, ZEN, DON: 0.4%	Almeida *et al.* [62]
	sows feed	FBs, ZEN	127	FB, ZEN: 1.5%	
EU (CZ, DK, ES, PT, HU)	sows feed, wheat, maize	23 mycotoxins	82	75% co-contaminated	Monbaliu *et al*. [58]
Slovakia	poultry feed	trichotecenes, ZEN	50	84% co-contaminated	Labuda *et al*. [71]
				DON, ZEN, T-2, HT-2: 32%	
		FB1, FB2, moniliformin		50% contained all three	Labuda *et al*. [70]
Netherlands	diet of dairy cattle	20 mycotoxins	169	DON and ZEN in 44% of the diets(major source: silage and compound feed)	Driehuis *et al*. [104]
Spain	barley	AFs, ZEN, OTA	123	AF, OTA: 31%	Ibáñez-Vea *et al*. [64]
				AF, ZEN: 12%	
				AF, ZEN, OTA: 27%	
		type A and B trichothecenes		43% contained ≥3 trichothecenes	Ibáñez-Vea *et al*. [65]
		combined:		AF, OTA, DON: 29%	Ibáñez-Vea *et al*. [105]
				AF, OTA, DON, ZEN: 26%	

The frequent detection of mycotoxin co-occurrence even in studies screening for a limited number of analytes underlines the importance of multi-mycotoxin analysis methods. Berthiller *et al.* [6] published a comprehensive review on the evolution of such methods from HPLC-UV to state of the art LC-MS/MS. Of the reports summarised above, Goertz *et al.* [57], Monbaliu *et al.* [58] and Driehuis *et al.* [104] used LC-MS/MS methods for the simultaneous detection of various mycotoxins. A very powerful method to this end was developed by Sulyok *et al.* who extended their LC-MS/MS method published in 2006 [106] to detect 87 mycotoxins [2]. Upon further development the method was extended by another 19 mycotoxins [107]. Today, it allows for the simultaneous detection of 340 analytes, most of them being mycotoxins (Personal Communication, M. Sulyok). 

## 7. Conclusions—Future Perspectives

Mycotoxins are ubiquitously present in European feed and feed raw materials. Although compliance with EU regulations is usually high, continuous monitoring is needed in order to avoid negative impacts on animal health and performance due to elevated contamination levels, which may occur for example after growing seasons characterised by weather conditions particularly favourable to fungal infection and growth. As the timing of the rainfall may be more important than the actual amount of precipitation [20,22], the development of predictive models for mycotoxin occurrence based on regional weather data would be a valuable tool to estimate the risk of contamination after a given growing season. For example, DONcast^®^, a predictive tool for DON in wheat in Ontario (Canada), has been commercialised in 2002 [108]. A European version (DONcast^®^ Europe) is under validation (www.doncast.eu). In Italy, a risk assessment tool predicting FB production by *F.* *verticillioides* in maize is under development [109]. Although the weather is the most influential parameter regarding the extent of mycotoxin contamination (it was found to explain 48% of the variation in DON concentration observed in wheat and maize in a Canadian study [110]) other factors are important as well. These are for example the choice of variety, crop rotation (avoiding maize as a pre-crop for wheat), tillage (ploughing reduces inoculum from plant residues), or the planting date (earlier planting of maize is generally preferable). Jouany [94] provided a comprehensive review on this subject. Some studies also investigated whether organically farmed products were more prone to mycotoxin contamination resulting from the absence of pesticide use. Conflicting results have been obtained on this issue, suggesting that other factors may override the effect of chemical control of fungal infection on the prevention of mycotoxin formation [94]. By the same token, there is no indication that organically farmed animal products should be considered any more or less risky in terms of mycotoxin contamination than those produced by conventional farming [111].

It is also necessary to increase the farmer’s awareness to the issue of mycotoxins in feed. Piva *et al.* [55] reported that northern Italian farmers had been warned about elevated AFB_1_-levels in maize after the 2003 growing season but that the warning was dismissed until the first detection of AFM_1_ in milk. The occurrence of AFB_1_ at such high levels in Europe also underlines the fact that climate change will entail a change in the mycotoxin distribution patterns observed today. 

With regard to the frequent detection of mycotoxin co-occurrence, even if only a very limited number of mycotoxins are analysed, and the evidence of possible additive or synergistic interaction of co-occurring mycotoxins, guidelines or maximum levels should not only be set for each mycotoxin individually but also for particularly concerning combinations which would definitely require more data on the impact of different mycotoxin combinations on different animals species. Possible synergistic interactions with so-called emerging mycotoxins such as moniliformin, beauvericin or enniatins should not be neglected in this respect. 

Although a lot of efforts to prevent mycotoxin formation have been undertaken, contaminations of those secondary fungal metabolites still occur. Therefore mycotoxin reduction strategies like the addition of mycotoxin deactivating products based on different strategies (adsorption, biotransformation, biodegradation, bioprotection) should be considered. Also, the development and application of multi-mycotoxin LC-MS/MS methods should be encouraged in order to get a more accurate picture of the extent of multi-mycotoxin contamination. These methods are especially valuable when it comes to detecting masked mycotoxins. Essentially, the term refers to conjugates of mycotoxins that typically go undetected when testing for the parent toxin. These conjugates may be produced by the fungus itself (3-AcDON, 15-AcDON) or formed as a part of the infected plant’s defence mechanism (DON-3-Glucoside, ZEN-4-Glucoside) [112]. Apart from exerting toxic effects themselves, there is evidence that some conjugates may be converted into the parent toxin during digestion, further adding to the toxicity of the feed [113,114]. 
